# Somatic CRISPR tumorigenesis and multiomic analysis reveal a pentose phosphate pathway disruption vulnerability in MPNSTs

**DOI:** 10.1126/sciadv.adu2906

**Published:** 2025-08-13

**Authors:** Gavin R. McGivney, Qierra R. Brockman, Nicholas Borcherding, Amanda Scherer, Adam J. Rauckhorst, Wade R. Gutierrez, Shane R. Solst, Collin D. Heer, Akshaya Warrier, Warren Floyd, David G. Kirsch, Vickie L. Knepper-Adrian, Emily A. Laverty, Grace A. Roughton, Douglas R. Spitz, Eric B. Taylor, Rebecca D. Dodd

**Affiliations:** ^1^ Department of Internal Medicine, University of Iowa Carver College of Medicine, Iowa City, IA 52240, USA.; ^2^ Holden Comprehensive Cancer Center, University of Iowa Carver College of Medicine, Iowa City, IA 52240, USA.; ^3^ Department of Molecular Physiology and Biophysics, University of Iowa Carver College of Medicine, Iowa City, IA 52240, USA.; ^4^ Department of Medicine, Anschutz Medical Campus, University of Colorado Cancer Center, Aurora, CO 80045, USA.; ^5^ Department of Pathology and Immunology, Washington University School of Medicine, St. Louis, MO 63110, USA.; ^6^ Fraternal Order of Eagles Diabetes Research Center (FOEDRC), University of Iowa Carver College of Medicine, Iowa City, IA 52240, USA.; ^7^ FOEDRC Metabolomics Core Research Facility, University of Iowa Carver College of Medicine, Iowa City, IA 52240, USA.; ^8^ Department of Urology, Mayo Clinic, Rochester, MN 55905, USA.; ^9^ Department of Radiation Oncology, Free Radical and Radiation Biology Program, University of Iowa Carver College of Medicine, Iowa City, IA 52240, USA.; ^10^ Department of Radiation Oncology, University of Texas MD Anderson Cancer Center, Houston, TX 77030, USA.; ^11^ Radiation Medicine Program, Princess Margaret Cancer Centre, University Health Network and the University of Toronto, Toronto, ON, Canada.; ^12^ Department of Radiation Oncology and Medical Biophysics, University of Toronto, Toronto, ON, Canada.; ^13^ Abboud Cardiovascular Research Center, University of Iowa Carver College of Medicine, Iowa City, IA 52240, USA.; ^14^ Pappajohn Biomedical Institute, University of Iowa Carver College of Medicine, Iowa City, IA 52240, USA.

## Abstract

Malignant peripheral nerve sheath tumors (MPNSTs) are aggressive and chemo-resistant sarcomas with poor survival rates. Loss of *CDKN2A* or *P53* following NF1 disruption is a key event in MPNST development. Here, we used CRISPR-Cas9 somatic tumorigenesis in mice to identify transcriptomic and metabolomic features distinguishing *CDKN2A*- versus *P53*-deleted MPNSTs. Convergent, multiomic analyses revealed that *CDKN2A*-deleted MPNSTs are especially dependent on the pentose phosphate pathway (PPP) and NADPH metabolism for growth and viability. Disruption of glucose-6-phosphate dehydrogenase (G6PD), the PPP rate-limiting enzyme, slowed *CDKN2A*-deleted MPNST growth and sensitized MPNSTs to standard-of-care chemotherapy. Knockdown of the redox-regulated transcription factor NRF2 slowed MPNST growth and decreased G6PD transcription. Analysis of patient MPNSTs identified a NRF2 gene signature correlating with tumor transformation. Furthermore, G6PD and NRF2 expression in PanCancer TCGA samples correlates with patient survival. This work identifies NRF2-PPP dependency as a targetable vulnerability in these difficult-to-treat MPNSTs, particularly in the *NF1/CDKN2A*-deleted majority.

## Teaser

Tumor suppressor status highly affects cellular reprogramming, creating genotype-specific sensitivities in MPNST.

## INTRODUCTION

Malignant peripheral nerve sheath tumors (MPNSTs) are painful, debilitating sarcomas of the myelinating nerve sheath. Approximately 70% of MPNSTs are unresectable or metastatic at diagnosis, and doxorubicin chemotherapy and radiation remain the standard of care for MPNSTs despite being largely ineffective (*1*, *2*). In patients with neurofibromatosis type 1 tumor predisposition syndrome, MPNSTs arise from plexiform neurofibromas. These benign precursor lesions develop following biallelic loss of the neurofibromin (*NF1*) gene. Transformation into MPNSTs requires secondary genetic disruptions that are frequently observed in the *CDKN2A* and *P53* genes. While *CDKN2A* alterations are the most common (reported at 38 to 80%), a smaller number of MPNSTs have genetic alterations in *P53* (reported at 17 to 50%) (*3*–*7*). Thus, deeper understanding of the *CDKN2A*- and *P53*-deficient MPNST subtypes may provide critically needed insight for developing new therapies.

The *CDKN2A* locus generates two distinct gene products—INK4A (p16) and ARF (p14 in humans and p19 in mice), which control multiple cell cycle pathways. Similarly, *P53* is a canonical cell cycle regulator. While there is substantial cross-talk between the *CDKN2A* and *P53* cell cycle pathways, these tumor suppressors have demonstrated cell cycle–independent roles in the production of and response to reactive oxygen species (ROS). Gene programs regulated by both tumor suppressors include genes induced by the master oxidant-responsive transcription factor NRF2 (*NFE2L2*). These NRF2-regulated ROS response pathways promote cancer cell survival, raising the possibility of metabolic vulnerabilities accompanying loss of *CDKN2A* or *P53*. Metabolic roles of *CDKN2A* and *P53* have been observed independently, but to our knowledge, comparative mechanistic studies have not been reported (*8*–*11*). Moreover, given the high frequency of *CDKN2A* and *P53* disruption in cancer, a deeper understanding of the molecular and biochemical effects of their role in MPNSTs may inform broader cancer mechanisms and potential therapies.

The overall goal of this study was to mechanistically define the distinctive roles of *CDKN2A* and *P53* loss in MPNSTs. To achieve this, we used CRISPR-Cas9 somatic tumorigenesis models of *Nf1*/*Cdkn2a*-deleted and *Nf1/p53*-deleted autochthonous MPNSTs in genetically identical, wild-type mice (*12*–*14*). Transcriptomic analysis of *Nf1/Cdkn2a*-deleted versus *Nf1/p53*-deleted tumor-derived cell lines revealed differential enrichment of multiple metabolic pathways, including for ROS responses. In addition, we conducted parallel metabolomic analysis and subsequent mechanistic molecular-genetic and biochemical tests, which corroborated RNA sequencing (RNA-seq) data. These data demonstrate that compared to *Nf1*/*p53*-deleted MPNSTs, *Nf1/Cdkn2a*-deleted MPNSTs exhibit a greater reliance on the NRF2-regulated ROS defense pathway, the pentose phosphate pathway (PPP). Disrupting the PPP sensitized both *Nf1/Cdkn2a*-deleted and *Nf1/p53*-deleted MPNSTs to standard-of-care chemotherapy. Last, human patient data showed NRF2/PPP gene set enrichment following tumor transformation, underscoring future therapeutic opportunities for metabolic targeting in MPNSTs.

## RESULTS

### Development of in vivo CRISPR-Cas9 models of MPNST tumor suppressor status

**Fig. 1.**
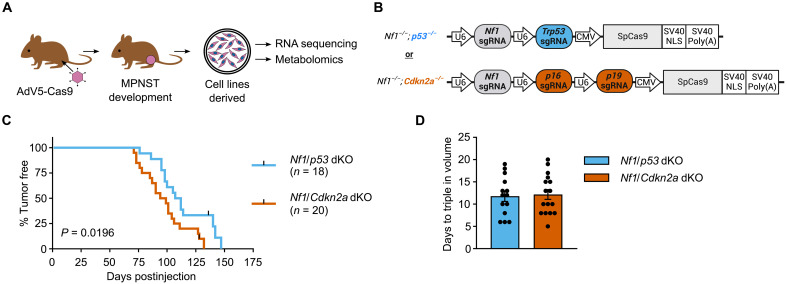
Derivation of primary tumor cell lines for direct comparison of tumor suppressor roles in MPNST. (**A**) Schematic of development of *Nf1*/*p53*-deleted (*Nf1*/*p53* dKO) and *Nf1*/*Cdkn2a*-deleted (*Nf1*/*Cdkn2a* dKO) murine orthotopic CRISPR-Cas9 primary MPNST cell lines for multiomic analysis. Schematic made using artwork from www.vecteezy.com (https://www.vecteezy.com/free-vector/mouse). (**B**) Ad5 CRISPR-Cas9 sgRNA viral constructs injected into sciatic nerve for primary MPNST tumorigenesis (A). Schematic made using Inkscape software. (**C**) Kaplan-Meier curve comparing primary tumor initiation from time of adenovirus injection (day 0) of 129 Sv/Jae murine strain injected with *Nf1*/*p53* AdV5-CMV-Cas9 (*n* = 18 biological replicates) and *Nf1*/*Cdkn2a* AdV5-CMV-Cas9 (*n* = 20 biological replicates). (**D**) Days for tumors [in (C)] to triple in volume postinitiation. Data presented as mean ± SEM. *P* value was determined by log-rank test [in (C)] and two-tailed unpaired *t* test [in (D)].

Here, we aimed to directly compare the roles of *CDKN2A* and *P53* in MPNST molecular-metabolic programming. To achieve this, we analyzed tumor-derived cell lines from *Nf1*/*Cdkn2a*-deleted or *Nf1*/*p53*-deleted MPNSTs obtained from genetically matched wild-type mice (Fig. 1A). Compared to prior models of MPNST tumorigenesis that have used genetically engineered mouse models, the in vivo somatic tumorigenesis approach applied here offers two key advantages. First, beginning with genetically identical wild-type mice avoids the confounding effects of difficult-to-detect genetic drift within separate genetically modified lines (*15*). Second, this approach is faster and less costly, especially when comparing multiple alleles (*12*). We began by validating an updated CRISPR-Cas9 system for peripheral nerve sheath tumorigenesis in 129 Sv/Jae mice. We used injection of adenovirus-containing cytomegalovirus (CMV)–driven, codon-optimized Cas9 endonuclease and guide RNAs directly into the sciatic nerve of immune-competent, wild-type mice (*16*). In experiments comparing the updated system to the prior model that used Cbh-driven Cas9, we observed similar rates of *Nf1*/*p53*-deleted tumor initiation in 129 Sv/Jae mice (fig. S1A) (*12*).

To begin directly comparing the effects of *p53* versus *Cdkn2a* loss, we next injected *Nf1*/*p53* or *Nf1*/*Cdkn2a*-targeting adenovirus into 129 Sv/Jae mice (Fig. 1B). As an additional step to validate the model, we also tested the tumorigenic constructs in wild-type C57/Bl6 mice. Tumor onset of *Nf1*/*p53*-deleted MPNSTs was similar between 129Sv/Jae and C57/Bl6 mice (fig. S1B) (*14*). Conversely, 129Sv/Jae mice developed *Nf1*/*Cdkn2a*-deleted tumors more quickly than C57/Bl6 mice (Fig 1C and fig. S1, C and D). In contrast to tumor onset, there was no difference in tumor growth rates across genotypes or murine strains, as indicated by the number of days required to triple in volume and day 10 tumor volumes (Fig. 1D and fig. S1, E and F). These data collectively suggest that while the differential loss of these tumor suppressors can influence tumor initiation, it does not affect overall tumor growth rates. Given the historical use of 129 Sv/Jae mice for the CRISPR-Cas9–based somatic tumorigenesis MPNST model and the absence of growth differences between129 Sv/Jae and C57/Bl6 MPNSTs, we continued with 129 Sv/Jae MPNST-derived cell lines for mechanistic studies.

### Transcriptomic analysis identifies global genotype-dependent differential regulation

**Fig. 2.**
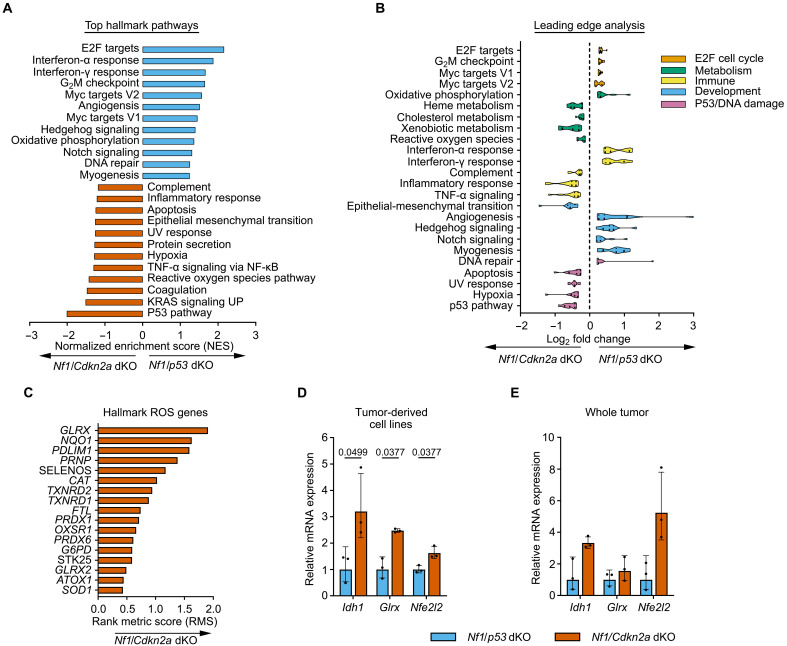
Differential tumor suppressor loss alters transcriptomes of MPNST. (**A**) RNA-seq analysis of primary cell lines: GSEA pathway enrichment analysis, top cancer hallmark pathways, (blue) *Nf1*/*p53* dKO (*n* = 3) versus (red) *Nf1*/*Cdkn2a* dKO (*n* = 3), each cell line run in triplicate. (**B**) Leading edge analysis of RNA-seq data. UV, ultraviolet; TNF-α, tumor necrosis factor–α; NF-κB, nuclear factor κB. (**C**) Top enriched genes in GSEA hallmark ROS gene set in *Nf1*/*Cdkn2a* dKO cell lines (*n* = 3). (**D** and **E**) RT-qPCR validation of RNA-seq (D) tumor-derived cell lines, *Nf1*/*p53* dKO (*n* = 3) and *Nf1*/*Cdkn2a* dKO (*n* = 3), and (E) whole tumor lysates, *Nf1*/*p53* dKO (*n* = 3) and *Nf1*/*Cdkn2a* dKO (*n* = 3). Data presented as geometric mean and geometric SD [in (D) and (E)], multiple unpaired *t* tests followed by Holm-Sidak correction [in (D) and (E)]. *P* = 0.05.

To investigate differences in transcriptional programming between *Nf1*/*Cdkn2a*- and *Nf1*/*p53*-deleted MPNST cells, we performed RNA-seq on cell lines derived from three independent tumors of each genotype (Fig. 1A). Expression analysis identified that 7143 transcripts were differentially expressed (adjusted *P* < 0.05) between the two genotypes. Gene set enrichment analysis (GSEA) demonstrated that *Nf1*/*p53*-deleted tumors were enriched for targets of E2F, a transcription factor downstream of the INK4A-retinoblastoma (RB1) tumor suppressor pathway (Fig. 2A). Similarly, *Nf1/Cdkn2a*-deleted tumors were enriched for genes in the p53 pathway, which is still intact in these tumors. These findings were complemented by leading edge analysis, which reports the core subset of transcripts contributing to the gene set’s enrichment signal (*17*). This analysis demonstrated that *Nf1*/*Cdkn2a*-deleted tumors were enriched in gene ontology pathways involved in “metabolism” and “p53/DNA damage” (Fig. 2B). In contrast, genes overrepresented in *Nf1*/*p53*-deleted cells included pathways such as “E2F cell cycle,” “DNA repair,” and “development.” Differential enrichment of immune-specific pathways occurred in both *Nf1*/*p53*-deleted and *Nf1*/*Cdkn2a*-deleted tumors. Of particular interest was the enrichment of ROS genes in *Nf1*/*Cdkn2a*-deleted tumors relative to *Nf1*/*p53*-deleted (Fig. 2C). Notably, increased expression of several ROS hallmark genes involved in antioxidant response—*Idh1*, *Nfe2l2* (NRF2), and *Glrx*—were validated by reverse transcription quantitative polymerase chain reaction (RT-qPCR) in both tumor-derived cell lines and whole tumor lysates (Fig. 2, D and E). These findings suggest that many hallmark cancer pathways are differentially regulated by loss of *CDKN2A* versus *P53* in MPNSTs.

### Multiomic analysis identifies unique therapeutic vulnerabilities in MPNSTs

**Fig. 3.**
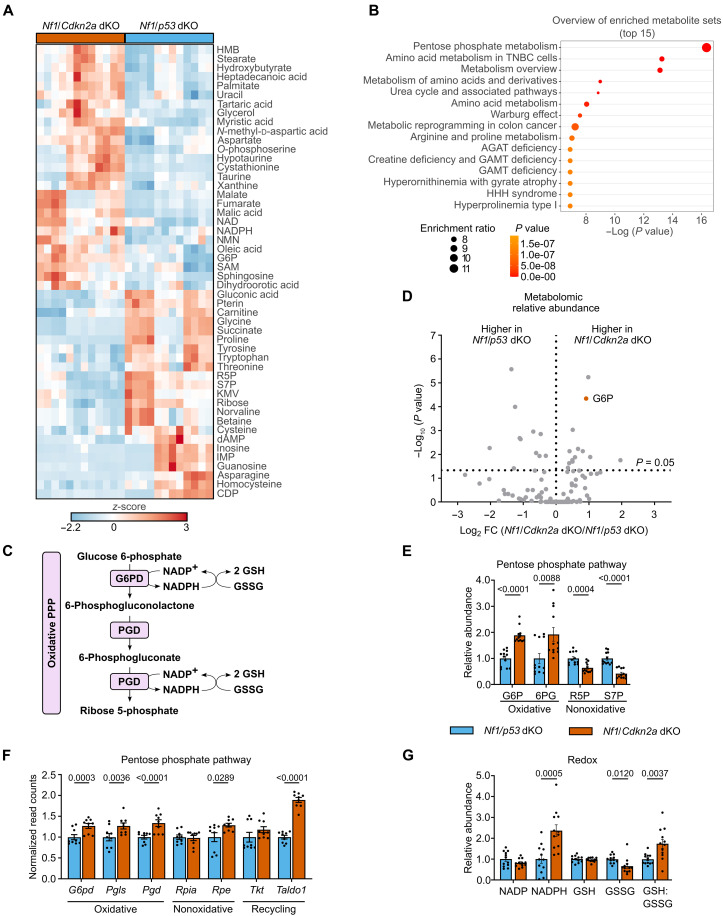
Loss of *Cdkn2a* results in enrichment of the PPP. (**A**) GC and LC metabolomics unsupervised clustered heatmap, scaling (*z*-score). IMP, inosine monophosphate; CDP, cytidine diphosphate; HMB, beta-hydroxy-beta-methylbutyrate; NMN, β-nicotinamide mononucleotide. (**B**) GC and LC metabolomics enriched metabolite set analysis. (**C**) Schematic of oxidative PPP:G6PD (glucose-6-phosphate dehydrogenase), PGLS (6-phosphogluconolactonase), PGD (6-phosphogluconate dehydrogenase), NADP^+^ (oxidized nicotinamide adenine dinucleotide phosphate), NADPH (reduced nicotinamide adenine dinucleotide phosphate), GSH (reduced glutathione), and GSSG (glutathione disulfide). TNBC, triple-negative breast cancer; AGAT, L-arginine:glycine amidinotransferase; GAMT, Guanidinoacetate methyltransferase; HHH, hyperornithinemia-hyperammonemia-homocitrullinuria. (**D**) Volcano plot showing interaction of fold changes (FC) and *P* values of metabolites, fold changes represent *Nf1*/*Cdkn2a* dKO mean relative to *Nf1*/*p53* dKO mean for each metabolite, G6P (glucose 6-phosphate), *P* = 0.05 indicated by a dotted line. (**E** and **G**) PPP and redox metabolites relative abundance, normalized to *Nf1*/*p53* dKO average for each metabolite. 6PG, 6-phosphogluconate; R5P, ribose 5-phosphate; S7P, sedoheptulose 7-phosphate; NADP, oxidized nicotinamide adenine dinucleotide phosphate. (**F**) PPP transcripts, RNA-seq read count values normalized to *Nf1*/*p53* dKO average for each transcript. *Nf1*/*p53* dKO (*n* = 3) and *Nf1*/*Cdkn2a* dKO (*n* = 3), each cell line run in quadruplicate (independent samples) [in (A) to (C), (E), and (G)] and triplicate (independent samples) [in (F)]. Data presented as mean ± SEM [in (E) to (G)]. Unpaired *t* tests [in (E) and (G)]. Adjusted *P* values were determined by differential expression analysis followed by Benjamini and Hochberg’s approach for false discovery rate (FDR) [in (F)]. *P* = 0.05.

Given the strong differential enrichment of metabolic and antioxidant pathways in RNA-seq data, the known importance of metabolism to tumor biology, and that metabolism cannot be directly observed via RNA-seq, we used metabolomics. We used both gas chromatography– and liquid chromatography–mass spectrometry (GC-MS and LC-MS) metabolomic profiling to examine relative metabolite abundances of *Nf1*/*Cdkn2a*-deleted and *Nf1*/*p53*-deleted MPNST cell lines derived from three independent tumors of each genotype. Unsupervised clustering heatmap analysis showed strong genotypic-specific differences in metabolism (Fig. 3A). Notably, 76 of the 141 metabolites identified were present at significantly different levels between the two genotypes. These metabolites represent multiple major metabolic pathways, including glycolysis, tricarboxylic acid cycle intermediates, essential and nonessential amino acids, and ribonucleotide synthesis (figs. S2 to S4 and data S1).

We further analyzed this dataset using quantitative metabolite set enrichment analysis. The most differentially enriched metabolite set was the PPP (Fig. 3B). The PPP plays an important role in cancer as a major source of reduced nicotinamide adenine dinucleotide phosphate (NADPH), a critical antioxidant that combats redox stress. NADPH is a central regulator of redox metabolism that supplies hydride to revert glutathione disulfide (GSSG) to reduced glutathione (GSH) and oxidized thioredoxin (TrxS2) to reduced thioredoxin [Trx(SH)_2_]. These processes constitute the major cofactors and reducing equivalents for hydroperoxide metabolic pathways in cancer cells through the activities of glutathione peroxidases and peroxiredoxins (*18*, *19*).

The PPP branches from glycolysis at the point of glucose 6-phosphate (G6P) and produces NADPH (*2*) from the enzymatic reactions of glucose-6-phosphate dehydrogenase (G6PD) and phosphogluconate dehydrogenase (PGD) (Fig. 3C). Corroborating the metabolite set enrichment analysis, secondary analysis of individual PPP metabolites showed that the metabolites G6P, 6-phosphogluconate, and NADPH were elevated in *Nf1/Cdkn2a*-deleted MPNSTs relative to *Nf1*/*p53*-deleted MPNSTs (Fig. 3, D, E, and G). Concordantly, RNA-seq data demonstrated higher expression of *G6pd* and other PPP transcripts in *Nf1*/*Cdkn2a*-deleted relative to *Nf1*/*p53*-deleted MPNSTs (Fig. 3F). Elevated levels of *G6pd* in the *Nf1*/*Cdkn2a*-deleted relative to *Nf1*/*p53*-deleted MPNSTs were further validated by RT-qPCR of whole tumor lysates (fig. S5A). Increased relative abundance of NADPH and higher ratio of GSH:GSSG provide further support for increased activity of the PPP (Fig. 3G). Furthermore, we observed increased expression of *Taldo1* transcript (Fig. 3F). Transaldolase 1 (*Taldo1*) aids in the recycling of nonoxidative PPP intermediates to fructose 6-phosphate, followed by conversion back to G6P. This cycling increases the yield from precursor glucose to efficiently meet high NADPH demands (*20*). Together, these data support the hypothesis that *NF1*/*Cdkn2a*-deleted MPNSTs up-regulated the PPP to enhance NADPH production relative to *Nf1*/*p53*-deleted MPNSTs.

### Loss of *Cdkn2a* results in PPP dependency

**Fig. 4.**
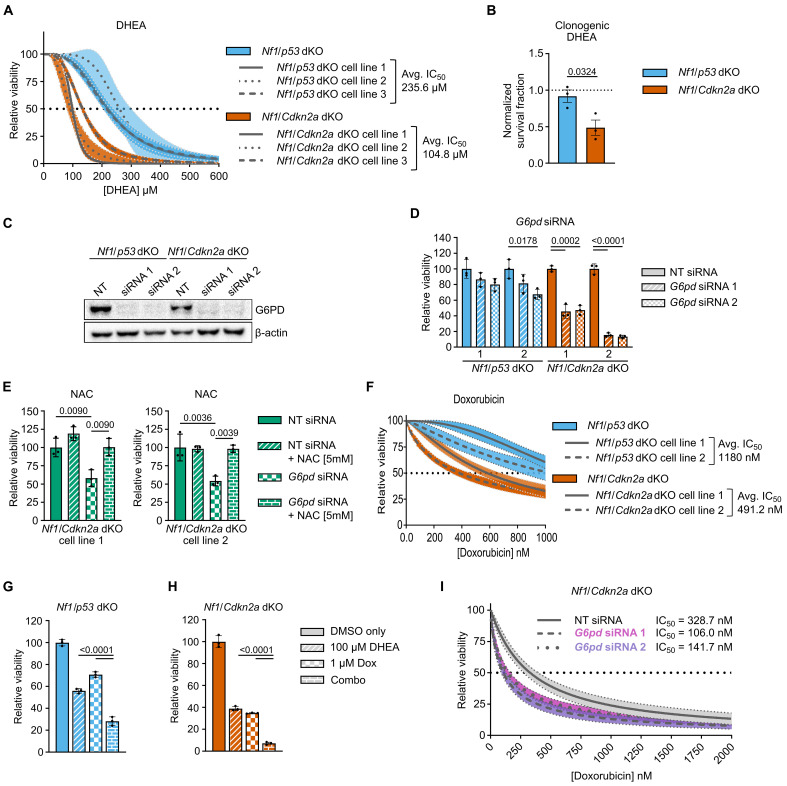
Loss of Cdkn2a results in increased vulnerability to PPP inhibition. (**A**) Representative dose-response curves of *Nf1*/*p53* dKO (*n* = 3) and *Nf1*/*Cdkn2a* dKO (*n* = 3) cell lines treated with DHEA for 48 hours. (**B**) Clonogenic survival assay post-DHEA (75 μM) treatment, each data point is the average of one cell line in triplicate, *n* = 3 per genotype, each cell line normalized to its own DMSO control. (**C**) Western blot of *Nf1*/*p53* dKO and *Nf1*/*Cdkn2a* dKO cell lines 72 hours posttransfection *G6pdx* targeted siRNA transfection, β-actin used as loading control. (**D**) Representative relative viability of cell lines 96 hours post-*G6pdx* targeted siRNA transfection. NT, nontargeting. (**E**) Representative relative viability of *Nf1*/*Cdkn2a* dKO cell lines 72 hours posttransfection with *G6pdx* targeted siRNA +/− 5 mM NAC. (**F**) Representative doxorubicin IC_50_ curves, *Nf1*/*p53* dKO (*n* = 2) and *Nf1*/*Cdkn2a* dKO (*n* = 2) cell lines treated for 48 hours. (**G** and **H**) Representative relative viability of (G) *Nf1*/*p53* dKO and (H) *Nf1*/*Cdkn2a* dKO treated with 100 μM DHEA, 1 μM Dox (doxorubicin), or combination for 48 hours. (**I**) Representative dose-response curves of *Nf1*/*Cdkn2a* dKO cells transfected with *G6pdx* targeted siRNAs and treated with doxorubicin 72 hours posttransfection for 48 hours. Curves fitted using nonlinear regression, option [inhibitor] vs normalized response-variable slope in GraphPad Prism. Shaded areas represent 95% confidence intervals [in (A), (F), and (I)], independently derived (different tumors) primary cell lines within each genotype represented by line patterns [in (A) and (F)], representing specific siRNA treatments [in (I)]. Genotype average IC_50_ and individual cell line IC_50_s denoted in (A), (E), and (I), IC_50_ confidence intervals shown in fig. S5 [for (A) and (F)]. Data presented as mean ± SEM, two-tailed unpaired *t* test [in (B)]. Data presented as mean ± SD, one-way analysis of variance (ANOVA) with Holm-Sidak correction [in (D), (E), (G), and (H)]. *P* = 0.05.

On the basis of these multiomic data, we hypothesized that PPP inhibition would decrease viability of *Nf1*/*Cdkn2a*-deleted MPNSTs more than *Nf1*/*p53*-deleted MPNSTs. To test this, we treated *Nf1*/*Cdkn2a*-deleted (*n* = 3) and *Nf1*/*p53*-deleted (*n* = 3) MPNST cells with the G6PD inhibitor dehydroepiandrosterone (DHEA) for 48 hours. The average median inhibitory concentration (IC_50_) for *Nf1*/*Cdkn2a*-deleted cells was >50% less than the average IC_50_ for *Nf1*/*p53*-deleted cells (mean IC_50_s of 104 μM versus 235 μM, respectively) (Fig. 4A and fig. S5B). Clonogenic assays also showed that *Nf1*/*Cdkn2a*-deleted MPNST cells were more sensitive to DHEA treatment (Fig. 4B). To complement these pharmacological tests, we genetically targeted *G6pd* with small interfering RNA (siRNA) (Fig. 4C). G6PD knockdown slowed the growth of both genotypes, with a markedly stronger effect in *Nf1*/*Cdkn2a*-deleted cells (Fig. 4D). Supplementation of the thiol antioxidant *N*-acetyl-l-cysteine (NAC) rescued the effects of G6PD knockdown in *Nf1*/*Cdkn2a*-deleted cells, consistent with rescue of oxidative stress caused by PPP disruption (Fig. 4E). Together, these data support the hypothesis that *Nf1*/*Cdkn2a*-deleted MPNSTs are uniquely more sensitive to PPP disruption.

MPNSTs are highly resistant to chemotherapy, with patients exhibiting only 25% response when treated with standard-of-care doxorubicin (Adriamycin). The MPNST cell lines showed differential, genotype-specific responses to doxorubicin. Average IC_50_s of doxorubicin in *Nf1*/*Cdkn2a*-deleted cells were ~480 nM, while values for *Nf1*/*p53*-deleted cells were over twofold higher (~1120 nM) (Fig. 4F and fig. S5C). To test whether G6PD inhibition could improve response to chemotherapy, we performed combination treatment studies. Notably, the combination of DHEA and doxorubicin proved to be more efficacious than either agent alone (Fig. 4, G and H). While this effect is more pronounced in *Nf1*/*Cdkn2a*-deleted versus *Nf1*/*p53*-deleted tumors, we note that the doxorubicin/DHEA combination was beneficial in both genotypes. Parallel studies with *G6pd* targeted siRNAs measured sensitization to doxorubicin in both MPNST genotypes. In *Nf1*/*Cdkn2a*-deleted cells, siRNA-induced knockdown of G6PD decreased the IC_50_ of doxorubicin from ~330 to <140 nM (Fig. 4I). In contrast, combining doxorubicin with *G6pd*-targeted siRNAs displayed no additive benefit in *Nf1*/*p53*-deleted cells (fig. S5D). Together, these results suggest that disrupting the PPP is detrimental to cells harboring *CDKN2A* inactivating mutations.

### The NRF2/G6PD axis drives *Nf1/Cdkn2a*-deleted MPNSTs

**Fig. 5.**
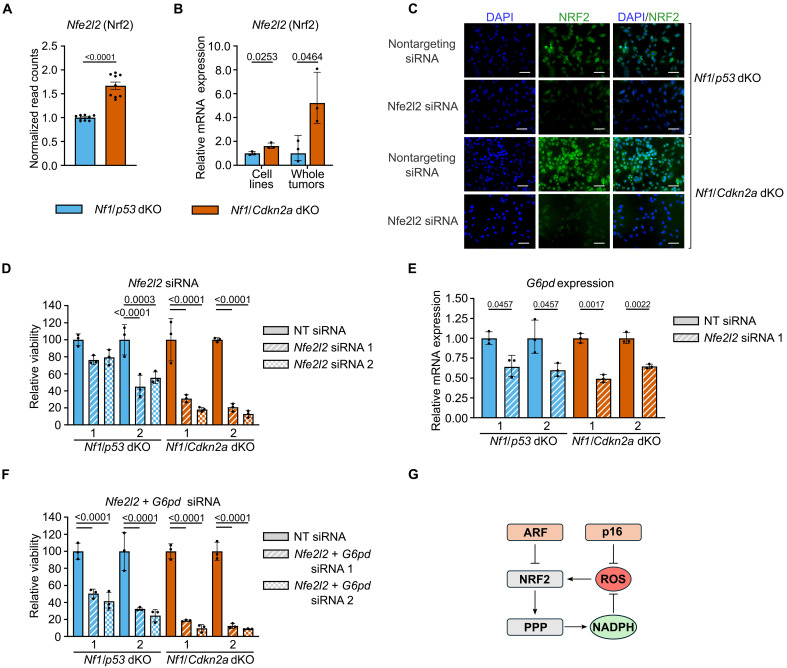
The NRF2/G6PD axis drives *Nf1/Cdkn2a*-deleted MPNST. (**A**) *Nfe2l2* transcript levels, RNA-seq read count values normalized to *Nf1*/*p53* dKO average. (**B**) RT-qPCR *Nfe2l2* transcript levels in *Nf1*/*p53* dKO and *Nf1*/*Cdkn2a* dKO cell lines and whole tumor lysates, normalized to *Nf1*/*p53* dKO average. (**C**) Immunofluorescence validation of NRF2 protein knockdown 72 hours posttransfection with *Nfe2l2* targeted siRNAs, representative images. Scale bars, 25 μm. DAPI, 4′,6-diamidino-2-phenylindole. (**D**) Relative viability of *Nf1*/*p53* dKO and *Nf1*/*Cdkn2a* dKO cell lines 96 hours posttransfection of *Nfe2l2* targeted siRNAs. (**E**) RT-qPCR, *G6pdx* transcript levels in cells 48 hours posttransfection of *Nfe2l2* targeted siRNA. (**F**) Relative viability of *Nf1*/*p53* dKO and *Nf1*/*Cdkn2a* dKO cell lines 96 hours postcombination transfection of *Nfe2l2* and *G6pdx* targeted siRNAs. (**G**) Model schematic of the proposed relationship between *Cdkn2a* gene products, ARF (p19) and p16, and the NRF2/PPP axis in regulating ROS levels in *Nf1*/*Cdkn2a*-deleted MPNSTs. Schematic made using Inkscape software. Data presented as mean ± SD, *Nf1*/*p53* dKO (*n* = 3) and *Nf1*/*Cdkn2a* dKO (*n* = 3), each cell line run in triplicate (independent samples), adjusted *P* values were determined by differential expression analysis followed by Benjamini and Hochberg’s approach for FDR [in (A)]. Data presented as geometric mean and geometric SD, Multiple unpaired *t* tests followed by Holm-Sidak correction [in (B) and (E)]. Data presented as mean ± SD, one-way ANOVA followed by Holm-Sidak correction [in (D) and (F)]. *P* = 0.05.

NRF2 (*NFE2L2*) is a transcription factor and master regulator of oxidant defense that increases expression of G6PD, PGD, and other NADPH-producing enzymes as well as glutathione- and thioredoxin-mediated hydroperoxide metabolic pathways (*21*). In addition, NRF2 has been shown to be negatively regulated by the *CDKN2A* gene product, ARF (*8*). To better understand the mechanism behind increased G6PD activity in *Nf1*/*Cdkn2a*-deleted MPNSTs, we examined our RNA-seq and subsequent RT-qPCR data. These data showed increased *Nfe2l2* transcript levels in *Nf1*/*Cdkn2a*-deleted MPNST cell lines and whole tumor lysates compared to the *Nf1*/*p53*-deleted MPNST cell lines (Fig. 5, A and B). In addition, we validated up-regulation of NRF2 targets by RT-qPCR in whole tumor and primary cell lines, including the redox gene *Txnrd1* (fig. S6, A and B). On the basis of results of G6PD inhibition, we hypothesized that *Nf1*/*Cdkn2a*-deleted cells would be more sensitive to *Nfe2l2* siRNA-mediated knockdown than *Nf1*/*p53*-deleted cells. NRF2 protein and *Nfe2l2* mRNA depletion were confirmed by immunofluorescence microscopy and RT-qPCR, respectively (Fig. 5C and fig. S6C). Similar to G6PD knockdown results, *Nf1*/*Cdkn2a*-deleted MPNST cells were more sensitive to NRF2 knockdown than *Nf1*/*p53*-deleted cells (Fig. 5D). Moreover, treatment with *Nfe2l2* targeting siRNAs also decreased *G6pd* mRNA levels by ~50%, supporting the role of NRF2 in G6PD regulation (Fig. 5E). We next tested combined *Nfe2l2* and *G6pd* siRNA targeted knockdown (Fig. 5F). While dual knockdown decreased growth of all cells more than either siRNA alone, the additive effect was stronger in *Nf1*/*p53*-deleted cells, which were much less affected by knockdown of single genes (Figs. 4D and 5D). These data suggest that an intact NRF2/G6PD axis is essential for normal MPNST viability, with *Nf1*/*Cdkn2a*-deleted cells being more dependent on it than *Nf1*/*p53*-deleted cells (Fig. 5G).

To further resolve the NRF2-dependent transcriptional program in MPNSTs, we evaluated other members of the NRF2 regulatory module. We did not observe differences in mRNA expression of the NRF2 regulatory proteins *Keap1* or *Cul3* between the two genotypes (fig. S6D). However, *Nf1*/*Cdkn2a*-deleted cells demonstrated increased mRNA abundance for the sMAF coactivators *Maff* and *Mafk* (fig. S6E). We also examined levels of other canonical NADPH-producing NRF2-regulated enzymes. Of these genes, we observed *Idh1* to have the greatest difference in mRNA levels between the *Nf1*/*Cdkn2a*-deleted and *Nf1*/*p53*-deleted cells (fig. S6F). We hypothesized that knockdown of *Idh1* in *Nf1*/*Cdkn2a*-deleted MPNSTs would have a similar effect on viability as *G6pd* or *Nfe2l2* siRNAs. However, while *Idh1* knockdown significantly decreased viability of *Nf1/Cdkn2a*-deleted cells, the effect was not as robust as *G6pd* or *Nfe2l2* knockdown (fig. S6, G and H). This suggests that IDH1 is not the main source of NADPH production in these tumors. Together, these results further support the vital role of the NRF2/G6PD axis in maintaining redox balance in MPNSTs.

### Patient MPNSTs have an active NRF2/G6PD axis

**Fig. 6.**
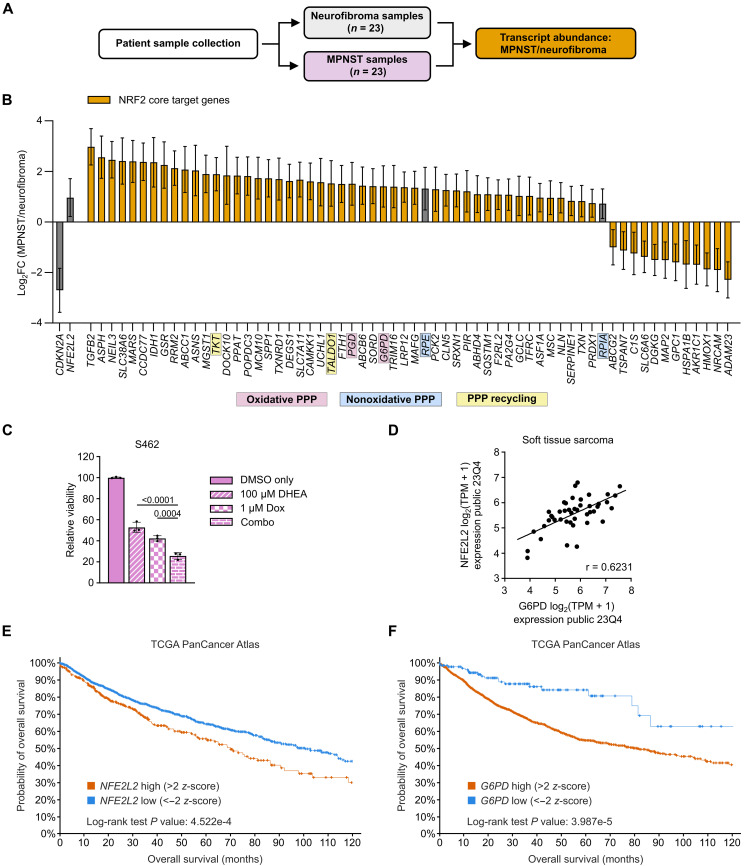
Patient MPNSTs have an active NRF2/G6PD axis. (**A**) Schematic: RNA-seq analysis of patient neurofibromas (*n* = 23) and MPNST (*n* = 23) samples. (**B**) NRF2 core target gene signature in human MPNST/neurofibroma RNA-seq dataset [from (A), NRF2 core target genes (orange), non-NRF2 core target genes (gray), oxidative PPP (highlighted in pink), nonoxidative PPP (highlighted in blue), and PPP recycling (highlighted in yellow)]. (**C**) Human MPNST cell line S462 treated with 100 μM DHEA, 1 μM doxorubicin, or combination for 48 hours. (**D**) *NFE2L2* and *G6PD* mRNA expression in soft tissue sarcoma cell lines. TPM, transcripts per million. (**E** and **F**) TCGA PanCancer Atlas, effect of mRNA expression levels on overall survival, *NFE2L2* high (*n* = 331) and low (*n* = 2426) [in (E)] and *G6PD* high (*n* = 1869) and low (*n* = 134) [in (F)]; high expression groups were determined by a *z*-score of >2, and low expression groups were determined by a *z*-score of <−2 [in (E) and (F)]. Data presented as mean log_2_ fold change ± SD, analyzed by mean MPNST expression compared to mean neurofibroma expression [in (B)]. Data presented as mean ± SD, one-way ANOVA followed by Holm-Sidak correction [in (C)]. Data analyzed using the Broad Cancer Dependency Map Project portal, each data point represents an individual cell line (*n* = 44), Pearson correlation (*r*) [in (D)]. Log-rank tests were conducted in cBioportal [in (E) and (F)]. *P* = 0.05.

We next aimed to determine whether there is evidence for NRF2/G6PD axis activation in human sarcomas and other cancers. First, we analyzed data from a published tissue microarray. This unique resource contains patient-matched MPNST progression samples including >20 benign neurofibromas and malignant tumor pairs with transcriptomic data from all patients. We compared the enrichment of gene expression between the aggregate MPNST samples and the benign neurofibromas (MPNST/neurofibroma ratio) (Fig. 6A). In concordance with established tumorigenesis pathways, our dataset showed significant loss of *CDKN2A* in MPNSTs relative to neurofibromas (Fig. 6B, in gray). We also observed up-regulation of NRF2 regulated oxidative PPP enzymes, *G6PD* and *PGD*, in MPNST samples (Fig. 6B, highlighted in purple). Similar to primary murine tumor and tumor-derived cell line data (Fig. 5, A and B), patient MPNSTs showed increased expression of *NFE2L2* (NRF2) (Fig. 6B, in gray). We next examined expression of a previously published NRF2 core target gene set and found that ~80% (48 of 60 genes) were enriched in MPNSTs relative to the neurofibromas (Fig. 6B, in orange). Following these notable results, we subjected human MPNST S462 cells having inactivating mutations in both *CDKN2A* and *P53* to DHEA and doxorubicin to evaluate the translational possibilities of this combination (*22*). Consistent with the mouse cell line results in Fig. 4 (F to H), the doxorubicin/DHEA combination decreased cell growth more than either agent alone (Fig. 6C). To expand the analysis to other sarcomas, we examined a large transcript expression dataset of patient-derived sarcoma cell lines. We identified strong correlations between NRF2 and G6PD expression in multiple sarcoma subtypes, including rhabdomyosarcoma, liposarcoma, synovial sarcoma, and MPNST. Combined, these data suggest the NRF2/G6PD axis may play a role in additional soft tissue sarcoma subsets (Fig. 6D and fig. S7, A to D). Last, we analyzed PanCancer The Cancer Genome Atlas (TCGA) data and observed a strong correlation between poor overall survival and high expression of either *NFE2L2* or *G6PD* (Fig. 6, E and F). Together, these results demonstrate activation of the NRF2/G6PD axis in MPNST development and identify a critical metabolic adaptation that MPNSTs undergo in transformation from a plexiform neurofibroma to an MPNST.

## DISCUSSION

Here, we show that MPNSTs harboring *Cdkn2a* or *p53* disruption secondary to loss of *Nf1* exhibit significant global transcriptional and metabolic differences. Multiomic and mechanistic analyses demonstrated a *Cdkn2a* loss–dependent up-regulation and critical metabolic vulnerability for the NRF2/G6PD axis. Moreover, we observe up-regulation of this axis during the transformation from benign neurofibroma to MPNST in human patient samples.

Both *CDKN2A* (INK4A/ARF) and *P53* play important roles in cancer metabolism but, to our knowledge, have not been directly compared. While INK4A canonically affects the RB1 cell cycle pathway, it also plays RB1-independent tumor suppressor roles, including negatively regulating accumulation of ROS and DNA damage (*9*, *10*). ARF, apart from its well-established role in attenuating mouse double minute 2 degradation of p53 (*23*–*25*), has P53-independent roles, including transcriptional repression of NRF2 in response to oxidative stress (*8*). In addition, P53 is known to regulate many metabolic pathways, including both ROS-promoting and ROS-inhibiting pathways (*11*). Despite both *CDKN2A* and *P53* having roles in the regulation of ROS, our data show that the *Cdkn2a*-deficient MPNSTs are more dependent on PPP activity for growth and chemoresistance.

Up-regulation of G6PD, the first and rate-limiting enzyme of the PPP, occurs in multiple cancers and increases NADPH production capacity, thereby playing a central role in protecting cells from ROS (*26*–*30*). In efforts to improve existing therapies, we showed that G6PD knockdown increases sensitivity to chemotherapy, a first-line treatment for metastatic MPNSTs and other soft tissue sarcomas. In addition, we identified greater activation of the upstream redox-regulated transcription factor NRF2 in *Nf1*/*Cdkn2a*-deleted MPNSTs relative to *Nf1*/*p53*-deleted MPNSTs. The transcription factor NRF2 functions as a tumor suppressor under normal physiological conditions by protecting cells from the protumorigenic effects of oxidative stress. However, hyperactivation of NRF2, due to mechanisms such as KEAP1 mutations or elevated NRF2 expression, can benefit cancer cells by protecting them from excessive oxidative stress and therapeutic agents, thereby promoting cancer cell survival (*31*, *32*). NRF2 regulates expression of multiple NADPH-producing enzymes, including G6PD, *PGD*, malic enzyme 1 (*ME1*), and isocitrate dehydrogenase 1 (*IDH1*). While we identified significant up-regulation of *Idh1* mRNA in *Nf1*/*Cdkn2a*-deleted tumors, knockdown of IDH1 had little impact on cell viability. This contrasts with the strong effect of G6PD knockdown in these cells and identifies a specific reliance on PPP-generated NADPH in these tumors. Other groups have reported similar observations in *KRAS* mutant colon cancer cells. In their studies, single and combinational knockouts of *G6PD*, malic enzyme 1, and isocitrate dehydrogenase 1 identify the PPP as the largest contributor to NADPH production, indicating these other enzymes function as “backups” in colon cancer, with little impact on NADPH production (*33*). Overall, our data suggest that not only is the PPP the primary source of NADPH in MPNST cells but that there is minimal supplementary NADPH production from alternative pathways.

Why does *Cdkn2a* loss, compared to *p53* loss, result in greater enrichment of and dependence on the NRF2/PPP axis? Conventionally, KEAP1 regulates NRF2 at the protein level by promoting NRF2 ubiquitination and subsequent proteasomal degradation (*34*). However, ARF can also regulate NRF2 activity by direct binding and sequestration of NRF2 that prevents it from regulating transcriptional programming (*8*, *35*). Therefore, loss of ARF could result in increased NRF2 activity. Similarly, as noted above, loss of INK4A can promote accumulation of ROS, leading to activation of NRF2. Thus, loss of *CDKN2A* promotes up-regulation of NRF2 transcriptional programming. Future studies are needed to distinguish the individual effects of p16 and ARF on NRF2 activity with loss of *CDKN2A*. The regulation of NRF2 by P53 alone is more complex, with potentially offsetting effects on transcript and protein abundance. P53 can decrease NRF2 transcript (*NFE2L2*) levels by inhibiting transcription factor SP1 from binding to its promotor region (*11*). Conversely, P53 can promote NRF2 protein stabilization via activation of p21 (*11*, *36*). In net, this supports the notion that loss of *Cdkn2a* promotes NRF2 transcription and activation, while loss of *P53* does not change or decrease NRF2 activity, creating a measurable difference in the transcriptional and metabolic reprogramming between these two genotypes.

Our results highlight translational opportunities. Patient MPNST samples showed transcriptional up-regulation of the NRF2/G6PD axis relative to benign precursor lesions, neurofibromas. Clinically used NRF2-specific inhibitors are now lacking. Although compounds such as brusatol, retinoic acid, and luteolin have demonstrated NRF2 pathway inhibitory effects, their translational potential is limited by issues such as toxicity, off-target effects, and context-dependent activity, including acting as NRF2 activators in certain settings (*37*). Upon further study and classification of these proposed NRF2 inhibitors, MPNSTs appear to be good candidates for future testing. In addition, as NRF2 is a master regulator, this outlines many potential downstream targets that promote accumulation of ROS when inhibited, including the PPP. Yet, inhibiting G6PD activity in the clinic has proven difficult. While DHEA was originally developed for clinical use, it is no longer used due to toxicities and multiple off-target activities. Recent encouraging progress has reported small-molecule inhibitors of G6PD activity that are being further developed for preclinical use (*38*). Nonetheless, our data suggest that future studies could examine alternative ROS-inducing inhibitors such as the thioredoxin reductase inhibitor, auranofin (*39*), proteosome inhibitors bortezomib and carfilzomib (*40*, *41*), or electron transport chain inhibitor, 2-methoxyestradiol (*42*), to attempt to increase chemotherapy response while lowering cumulative doses and toxicities. Ultimately, these data identify reliance on NADPH metabolism and the NRF2/G6PD axis in MPNSTs that represents a critical metabolic vulnerability for future therapeutic targeting in these aggressive cancers.

Our approach has advantages and limitations. One major advantage is the CRISPR-Cas9 method that delivers adenovirus-containing Cas9 and multiple guide RNAs into the sciatic nerve of mice to generate spatially controlled, autochthonous tumors that faithfully mimic human MPNSTs (*12*). By using this highly controlled system to compare distinct genetic mutations, we can simplify pathway comparisons and minimize confounding genetic events that can occur with previously used genetically engineered mouse models (*43*, *44*). A current limitation of our model is the absence of Cas9-directed induction of primary in vivo neurofibromas to directly compare MPNSTs to the pathological precursor lesion seen in patients with NF1. However, the human RNA-seq dataset provided corroborating insight into the metabolic reprogramming that occurs between the benign and malignant patient tumors. In addition, multiomic analyses were performed on tumor-derived cell lines in lieu of primary whole tumors. Nontransformed cells, including infiltrating fibroblasts and immune cells, do not remain viable during cell line derivatization. This process allowed for direct genetic comparison of the MPNST cells. Future exploration of the transcriptome and metabolome in whole tumors would add additional insight into the role of nontransformed cells on MPNST biology.

In summary, *Nf1*/*Cdkn2a*-deleted MPNSTs have an increased dependency on NADPH, which is met by elevated production through the PPP. Unlike *Nf1*/*Cdkn2a*-deleted MPNSTs, *Nf1*/*p53*-deleted MPNSTs were not as vulnerable to G6PD inhibition. However, G6PD inhibition improved response to chemotherapy in both genotypes. This suggests that single agent inhibition of G6PD may not be as efficacious for both tumor genotypes. Yet, overall, addition of G6PD inhibition to conventional chemotherapy could provide benefits for MPNST treatment and warrants further exploration.

## MATERIALS AND METHODS

### Animals and primary MPNST model development

To engineer CRISPR-Cas9–driven tumor models containing CMV promoters for Cas9, single guide RNAs (sgRNAs) targeting NF1, p16, and p19 (table S1) were assembled together with individual U6 promoters and sgRNA scaffold sequences using the Multiplex CRISPR-Cas9 Assembly System (Addgene, kit #1000000055) (*45*). After assembly into the A1x3 vector, the three sgRNAs were cloned into the Ad5CMVspCas9 shuttle plasmid (University of Iowa Viral Vector Core, #G1272) as a single piece of DNA using traditional restriction enzyme cloning (Cla I and Mlu I sites). Adenoviruses were produced at the University of Iowa Viral Vector Core as previously reported. Before injection, virus was mixed with Dulbecco’s modified Eagle’s medium (DMEM) and calcium phosphate as previously described (*46*). To induce tumorigenesis, 25 μl of prepared virus was injected into the left flank sciatic nerve of wild-type mice. Tumor initiation was declared at a measurable volume of ~150 to 200 mm^3^. Tumors were measured three times per week using a caliper. Tumor volumes were calculated using the formula *V* = (π × *L* × *W* × *H*)/6, with *L*, *W*, and *H* representing the length, width, and height of the tumor in mm, respectively. Tumors were harvested at a terminal size of 1500 mm^3^ or earlier if animals showed signs of distress in accordance with University of Iowa Institutional Animal Care and Use Committee guidelines. Tissue was collected for histology, RNA, and generation of cell lines.

### Derivation of cell lines

As previously published (*14*), tumor tissue was rinsed with 1× phosphate-buffered saline (PBS) and finely minced with dissection scissors in a six-well plate. Five milliliters of dissociation buffer [collagenase type IV (700 U/ml; Gibco, 17104-019) and dispase (65 mg/ml; Gibco, 17105-041)] was added to each well. The plate was incubated at 37°C for 1 hour on an orbital shaker and transferred to tissue culture hood. Tissue slurry was pipetted through a 70-μm cell strainer (Fisherbrand) and collected in a 50-ml conical tube. Cell strainer was washed with 25 ml of 1× PBS and collected in the same 50-ml conical tube. During this wash, the plunger from a 1-ml syringe (BD, 309628) was used to aid in collecting as much material as possible. The filtered suspension was centrifuged, and the supernatant was decanted. Cell pellets were resuspended in DMEM [(Gibco, 11965-092), +10% fetal bovine serum (R&D Systems, S11150), 1% penicillin-streptomycin (Gibco, 15140-122), and 1% sodium pyruvate (Gibco, 11360-070)] and plated in two wells of a six-well plate. Once 80 to 90% confluent, 33% of cells were transferred to a 10-cm dish. Cells were passaged at least 20 times before experimental analyses to ensure pure tumor populations.

### Metabolite extraction

Cell culture plates were washed twice with ice-cold PBS followed by two washes with ice-cold water before being flash frozen using liquid nitrogen. Cell culture plates were lyophilized overnight and then scraped into 1 ml of ice-cold 2:2:1 methanol/acetonitrile/water containing a mixture of internal standards (D4-citric acid, D4-succinic acid, D8-valine, and U13C-labeled glutamine, glutamic acid, lysine, methionine, serine, and tryptophan; Cambridge Isotope Laboratories) to extract metabolites. The mixtures were transferred to a microcentrifuge tube and flash frozen in liquid nitrogen, thawed for 10 min in a water bath sonicator, and rotated for 1 hour at −20°C. Mixtures were centrifuged for 10 min at 21,000*g*, and 300 μl of the cleared metabolite extracts was transferred to autosampler vials and dried using a SpeedVac vacuum concentrator (Thermo Fisher Scientific).

### GC-MS analysis

For GC-MS analysis, dried metabolite extracts were reconstituted in 20 μl of methoxyamine (11.4 mg/ml) in anhydrous pyridine, vortexed for 5 min, and heated for 1 hour at 60°C. Next, 16 μl of *N*,*O*-bis(trimethylsilyl)trifluoroacetamide was added to each sample, which was vortexed for 1 min and heated for 30 min at 60°C (*47*, *48*). One microliter of derivatized sample was injected into a Trace 1300 GC (Thermo Fisher Scientific) fitted with a TraceGold TG-5SilMS column (Thermo Fisher Scientific) operating under the following conditions: split ratio = 20:1, split flow = 24 μl/min, purge flow = 5 ml/min, carrier mode = constant flow, and carrier flow rate = 1.2 ml/min. The GC oven temperature gradient was as follows: 80°C for 3 min, increasing at a rate of 20°C/min to 280°C, and holding at a temperature at 280°C for 8 min. Ion detection was performed by an ISQ 7000 mass spectrometer (Thermo Fisher Scientific) operated from 3.90 to 21.00 min in electron ionization mode (−70 eV) using select ion monitoring.

### LC-MS analysis

For LC-MS (*47*), dried extracts were reconstituted in 20 μl of acetonitrile/water (1:1, v/v), vortexed well, rotated on a rotator in −20°C overnight, and centrifuged, and the supernatant was transferred to LC-MS autosampler vials for analysis. LC-MS data were acquired on a Thermo Q Exactive hybrid quadrupole Orbitrap mass spectrometer with a Vanquish Flex Ultra-High Performance Liquid Chromatography (UHPLC) system or Vanquish Horizon UHPLC system. The LC column used was a Millipore SeQuant ZIC-pHILIC (2.1 mm by 150 mm, 5-μm particle size) with a ZIC-pHILIC guard column (20 mm by 2.1 mm). The injection volume was 2 to 5 μl. The mobile phase comprised buffer A [20 mM (NH_4_)_2_CO_3_ and 0.1% NH_4_OH (v/v)] and buffer B (acetonitrile). The chromatographic gradient was run at a flow rate of 0.150 ml/min as follows: 0- to 21-min linear gradient from 80 to 20% buffer B; 20-to 20.5-min linear gradient from 20 to 80% buffer B; and 20.5- to 28-min hold at 80% buffer B. The MS operated in positive or negative timed single ion monitoring mode with the spray voltage set to 3.0 kV, the heated capillary held at 275°C, and the heated electrospray ionization probe held at 350°C. The sheath gas flow was set to 40 U, the auxiliary gas flow was set to 15 U, and the sweep gas flow was set to 1 U. MS data resolution was set at 70,000 and the automatic gain control target at 1 × 10^−6^ and a maximum injection time of 200 ms.

### GC-MS/LC-MS data analysis

Raw data were analyzed using TraceFinder (4.1 and 5.1) (Thermo Fisher Scientific). Targeted metabolites were identified on the basis of the University of Iowa Metabolomics Core facility standard-confirmed, in-house library defining a target ion and at least one confirming ion and retention time (GC) or accurate mass, retention time, and MS/MS fragmentation pattern when present (LC). For metabolomic profiling, a pooled sample was analyzed at the beginning, at a set interval during, and at the end the analytical run to correct for instrument drift using the NORmalization and EVAluation of Metabolomics

Data (NOREVA) tool (*49*). NOREVA-corrected individual metabolite values (arbitrary units) were then normalized: first, per sample, to the sum of identified metabolite values to control for extraction, derivatization, and/or loading effects; and second, across samples, dividing individual metabolite values by the average of that metabolite in a reference condition to set the reference condition to 1 while preserving error and to render metabolite values by fold of the reference. Metabolomic data are provided in excel sheet format (Data_S1_metabolomics_data), and RAW data files are available at the National Institutes of Health Common Fund’s National Metabolomics Data Repository (NMDR) website, the Metabolomics Workbench (study ID: ST003926, project DOI: http://dx.doi.org/10.21228/M8SG17) (*50*). Further data analysis and visualization was conducted using MetaboAnalyst (https://metaboanalyst.ca/). Heatmap settings were as follows: standardization (autoscale features), distance measure (Euclidean), and clustering method (Ward).

### RNA sequencing

Three primary murine MPNST cell lines of each genotype [*Nf1^−/−^; p53^−/−^* double-knockout (dKO), and *Nf1^−/−^; Cdkn2a^−/−^* dKO] were plated in triplicate (*n* = 3 per cell line) and collected with 500-μl TRIzol reagent (Ambion by Life Technologies, reference #15596018). Samples were purified using a Zymo Research Direct-zol RNA Miniprep kit (catalog no. R2052). After nanodropping, samples with low 260/280 and 260/230 were subjected to a Monarch RNA clean-up kit (50 μg) (catalog no. T2040S). RNA library preparation, transcriptome sequencing, and data analysis were performed by Novogene Co. Ltd. (Beijing, China). Gene expression data has been deposited to the GEO repository (Accession number: GSE275277). GSEA pathway analysis (*17*) was conducted with GSEA Hallmarks.v.7.1 gene sets (https://data.broadinstitute.org/gsea-msigdb/msigdb/release/7.1/h.all.v7.1.symbols.gmt).

### Reverse transcription quantitative polymerase chain reaction

As previously described (*51*), RNA was collected from six-well plated cells with 500-μl TRIzol reagent (Ambion by Life Technologies, reference #15596018). The Bio-Rad iScript cDNA Synthesis Kit (catalog no. 1708891) was used for cDNA production. RT-qPCR was performed with PowerUP Sybr Green 2× master mix (Thermo Fisher Scientific, A25778) per the manufacturer’s instructions on a QuantStudio-7 Flex or QuantStudio-7 Pro machine by the Genomics Division of the Iowa Institute of Human Genetics, University of Iowa. Analysis was performed with QuantStudio Design and Analysis software using the comparative Ct relative to β-actin or B2M RNA expression.

### Clonogenic cell survival

MPNST cells were counted and seeded in 60-mm tissue culture dishes at a density of 75,000 cells. Cells were plated 24 hours before treatment to allow for adherence and reentry to the cell cycle. DHEA treatment (75, 150, or 300 μM) was for 48 hours. Then, cells were trypsinized with 0.25% trypsin-EDTA (1×) (Thermo Fisher Scientific, catalog no. 25200056), collected, counted using a Coulter particle counter, and seeded into six-well tissue culture plates at a range of 200 to 5000 cells. Cells were returned to the incubator for 8 days to allow for colony formation. Colonies were fixed with 70% ethanol, stained with Coomassie Brilliant Blue G-250 (VWR International, CAS: 6104-58-1), and counted if there were >50 cells present. Cell survival for each treatment dose was calculated on the basis of the plating efficiency of the control plates for each cell line.

### siRNA phenotype studies

Similar to previous studies (*51*), transfection efficiency was assessed via flow cytometry following transfection of cells in 96-well plates (CELLSTAR, 655180), with TYE-563 fluor [Integrated DNA Technologies (IDT), 51-01-20-19] using a Jet PRIME transfection kit per the manufacturer’s instructions (101000046). All siRNA constructs were selected from the IDT website. For transfection experiments, cells were transfected with either a nontargeting control construct (50 nM) or constructs targeting *G6pdx* or *Nfe2l2* (single, 50 nM) per the manufacturer’s instructions. For validation of mRNA knockdown of each target, cell plating was scaled for a six-well (CELLSTAR, 657160) format based on a well surface area. Forty-eight hours posttransfection, transcript expression was analyzed via RT-qPCR (see the “Reverse transcription quantitative polymerase chain reaction” section). The 96-well siRNA phenotype studies were analyzed 96 hours posttransfection via Promega CellTiter-Glo (G7572) per the manufacturer’s instructions. For *G6pdx* siRNA phenotype rescue, [5 mM] NAC (Alfa Aesar, A15409, lot: U17G026) was coadministered with transfection of nontargeting or *G6pdx* siRNA. Twenty-four hours posttransfection, media was replaced with media alone or media + [5 mM] NAC. Plates were analyzed at 72 hours posttransfection. One molar stock of NAC was reconstituted fresh on day of use. A total of 0.4896 g of NAC was added to 0.2840 g of sodium bicarbonate (NaHCO_3_) (Thermo Fisher Scientific, S233). Three milliliters of deionized water was slowly added while on a stir plate, and pH was adjusted to 7.0. The final solution was filter-sterilized (0.22 μm) in tissue culture hood and further diluted to desired concentration in media. To analyze via CellTiter-Glo, phenotype experiments were conducted using white opaque 96-well plates (Greiner CELLSTAR, M0187).

### Immunofluorescence

For plating, 4375 cells per well were plated in Lab-Tek II chamber slides (154941). On day 2, cells were transfected with the siRNA constructs. Twenty-four hours posttransfection, media was replaced with fresh media. On day 4, cells were treated for 4 hours with 3 μM proteosome inhibitor (MG132, Cell Signaling, #2194). MG132 was reconstituted in dimethyl sulfoxide (DMSO; Sigma-Aldrich, D2438) and stored at −20°C in 10 mM aliquots. For fixation, media was aspirated off. Cells were then covered in 2 to 3 mm of 4% formaldehyde (Polysciences, #18814 16% formaldehyde) diluted in 1× PBS for 15 min at room temperature. Formaldehyde was then replaced with blocking buffer [1× PBS/5% goat serum (Sigma-Aldrich, #G9023)/0.3% Triton X-100 (Fisher Bioreagents, BP151-500)] for 1 hour at room temperature. Blocking buffer then replaced with primary antibody (Ab; NRF2, Cell Signaling Technology, #12721; 1:200) diluted in Ab dilution buffer [1× PBS/1% bovine serum albumin (Research Products International, #A30075-25.0)/0.3% Triton X-100] and incubated overnight at 4°C. Cells were then washed (three times) in 1× PBS for 5 min each. Cells were incubated for 1 to 2 hours at room temperature in fluorochrome-conjugated secondary Ab (1 μl/ml; goat anti-rabbit 488, Invitrogen, #A11070) diluted in Ab dilution buffer (see above). Cells were washed (three times) in 1× PBS for 5 min each. Slides were cover-slipped with ProLong diamond antifade mountant with 4′,6-diamidino-2-phenylindole (Invitrogen, P36962). Slides were cured overnight at room temperature. Cells were imaged with an inverted Olympus IX83 microscope with a 20× objective (HC PL APO CS2; 0.75 numerical aperture).

### In vitro inhibitor studies

For in vitro chemical inhibitor studies (DHEA, palbociclib, and doxorubicin), cells were plated in 96-well plates (1000 cells per well) (CELLSTAR, 655180 or Greiner CELLSTAR, M0187) and treated with desired chemical inhibitor concentrations. DHEA (dehydroisoandrosterone, Sigma-Aldrich, D4000) was reconstituted fresh for each experimental replicate. Palbociclib hydrochloride (MedChemExpress, #HY-50767A\CS-1327) was reconstituted in water (25 mM aliquoted stocks stored at −80°). Doxorubicin hydrochloride (MedChemExpress, #HY-15142/CS-1239) was reconstituted in water (50 mM aliquoted stocks stored at −80°). Experimental dilutions for chemical inhibitors reconstituted in DMSO were also conducted in DMSO, resulting in equal volumes of DMSO per chemical inhibitor concentration. Cells were treated by palbociclib for 72 hours. Cells were treated by DHEA or doxorubicin (or in combination) for 48 hours. Postdrug treatment, cells were analyzed via resazurin (DHEA and palbociclib) or Promega CellTiter-Glo (doxorubicin or doxorubicin + DHEA). Resazurin (Sigma-Aldrich, R7017) dissolved in PBS (1.5 mg/ml) was added to wells (20 μl per well for 96-well plates), and cells were returned to the tissue culture incubator for 1 to 2 hours before being read on a microplate reader (BioTek Instruments Synergy HT with GEN5 2.01 software) (*52*). Promega CellTiter-Glo (G7572) was added to each well according to the manufacturer’s protocol and returned to the incubator for 30 min before being read on a luminescence plate reader (Molecular Devices SpectraMax i3, Softmax Pro 6.5.1 software).

### Western blots

Cells were washed (two times) with 1× PBS and subsequently lysed with 1× Laemmli sample buffer (Bio-Rad, 1610747) as previously described (*52*). Wells were scraped (Biologix, catalog no. 70-1250), and lysates were transferred to microcentrifuge tubes. Lysates were heated to 95°C for 10 min and then sheared delicately to avoid bubbles with a 25-gauge needle and syringe. Lysates were centrifuged for 20 min at 16,000*g* at room temperature. The supernatant was transferred to new tubes and stored at −80°C. For future uses, samples were thawed on ice and heated to 70°C. Protein quantification was performed using Pierce 660 nM Protein Assay Reagent (Thermo Fisher Scientific, 22660) and neutralizer (G-Biosciences, 786-604) according to the manufacturers’ instructions. Equal amounts of protein were loaded in NuPAGE 4 to 12% bis-tris gels (Invitrogen, NP0335/NP0336). Gels were run at 50 V for 20 min and 120 V for 90 min using NuPAGE MES SDS Running Buffer (Novex, NP0002). Samples were transferred to polyvinylidene difluoride (Millipore, IPFL00010) at 20 V for 1 hour in NuPAGE Transfer Buffer (Novex, NP0006-1). Blots were blocked in 5% milk [1 g of powdered milk in 10 ml of tris-buffered saline (Bio-Rad, 1706435) with 0.1% Tween (TBS-T)] for 1 hour at room temperature. Blots were rinsed (three times) with TBS-T and then incubated in primary Ab in TBS-T for 24 hours at 4°C on a VWR rocking platform (VWR 10860). Blots were washed in TBS-T three times for 10 min each and then incubated in horseradish peroxidase (HRP)–conjugated secondary Ab in TBS-T for 1 hour at room temperature. Blots were again washed in TBS-T three times for 10 min each. Blots were imaged using a ChemiDoc (Bio-Rad XRS+, ImageLab 4.1 software) and SuperSignal West Pico PLUS Chemiluminescent Substrate (Thermo Fisher Scientific, 34577). The primary Abs used were anti-G6pdx (Cell Signaling Technology, #8866; 1:1000) and anti–β-actin (Cell Signaling Technology, #4970; 1:15,000). The secondary Ab used was goat anti–rabbit HRP (Jackson ImmunoResearch Laboratories, 111-035-144).

### Analysis of human patient datasets

Construction of the tissue microarray with matched MPNST/neurofibroma patient samples and gene expression analysis has been previously described (*53*). Briefly, total RNA was extracted from FFPE MPNST/neurofibroma patient samples, sequenced on the Illumina HiSeq 4000 genome sequencer (Illumina) at the University of Iowa Institute of Human Genetics, Genomics Division (Iowa City, IA), and pseudo-aligned using Kallisto (GSE145064) (*54*). For this study, we used the aligned count matrices from individual patients and converted ensembl transcript IDs to gene symbols with BioMart. Then, expression of CDKN2A, PPP genes, and an NRF2 core target gene set were compared by sample type (*55*). The TCGA PanCancer Atlas dataset was analyzed in cBio Portal to evaluate mRNA expression levels (*z*-score = ±2.0) of *NFE2L2* and *G6PD* in correlation with overall survival (*56*, *57*). Correlation of *NFE2L2* and *G6PD* expression in soft tissue sarcoma cell lines was analyzed using DepMap, Broad (2023). DepMap 23Q4 Public, Figshare+, dataset; https://doi.org/10.25452/figshare.plus.24667905.v2.

### Statistics

GraphPad Prism 10.2.3 and Excel were used for statistical analysis. Significance was determined by *P* < 0.05. Data were analyzed using two-tailed unpaired *t* test (two groups) or one-way analysis of variance (ANOVA) followed by Holm-Sidak correction (three or more groups). Kaplan Meier survival plots were analyzed by a log-rank (Mantel-Cox) test. IC_50_ curves were fitted using nonlinear regression, option “[inhibitor] vs normalized response-variable slope” in GraphPad Prism. Shaded areas represent 95% confidence intervals. Correlation plot data were analyzed with a Pearson correlation test (*r*). RNA-seq statistical analysis was conducted by Novogene. Differential expression analysis between two conditions/groups (two biological replicates per condition) was performed using the DESeq2 R package (1.14.1). The resulting *P* values were adjusted using the Benjamini and Hochberg’s approach for controlling the false discovery rate (FDR). Genes with an adjusted *P* < 0.05 found by DESeq2 were assigned as differentially expressed.
