# Supplementary material for: Somatic CRISPR tumorigenesis and multiomic analysis reveal a pentose phosphate pathway disruption vulnerability in MPNSTs
Source: Sci Adv. 2025 Aug 13;11(33):eadu2906. doi: 10.1126/sciadv.adu2906 (PMC12346278; doi:10.1126/sciadv.adu2906)
Supplement: Supplementary file 1 — Figs. S1 to S7 Table S1 Legend for dataset S1 [file sciadv.adu2906_sm.pdf]

Science Advances
AAAS

Supplementary Materials for

Somatic CRISPR tumorigenesis and multiomic analysis reveal a pentose phosphate pathway disruption vulnerability in MPNSTs

Gavin R. McGivney *et al.*

Corresponding author: Rebecca D. Dodd, rebecca-dodd@uiowa.edu; Eric B. Taylor, eric-taylor@uiowa.edu

Sci. Adv. **11**, eadu2906 (2025)
DOI: 10.1126/sciadv.adu2906

The PDF file includes:

Figs. S1 to S7
Table S1
Legend for dataset S1

Other Supplementary Material for this manuscript includes the following:

Dataset S1

Fig. S1.

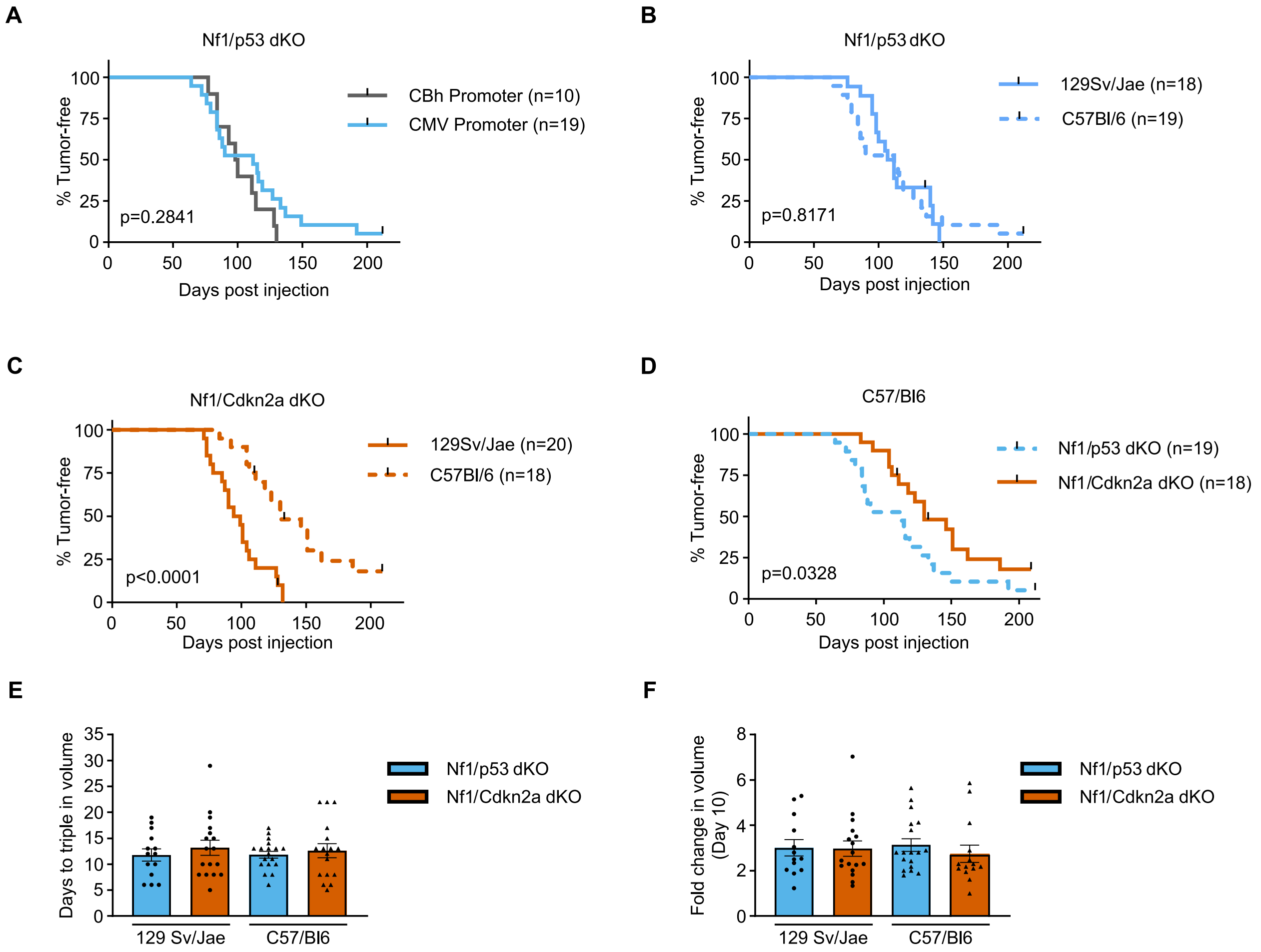

Tumor suppressor status impacts tumor onset, but not tumor growth, in a strain-dependent manner. (**A-D**) Kaplan Meier plots comparing tumor initiation from time of adenovirus injection (Day 0) of (**A**) *Nf1/p53* dKO primary tumors developed by CBh (n = 10 biological replicates) and CMV (n = 19 biological replicates) promotor driven Cas9 viral constructs, (**B**) 129 Sv/Jae (n = 18 biological replicates) and C57 Bl/6 (n = 19 biological replicates) murine strains injected with *Nf1/p53* Adv5-CMV-Cas9, (**C**) 129 Sv/Jae (n = 20 biological replicates) and C57 Bl/6 (n = 18 biological replicates) murine strains injected with *Nf1/Cdkn2a* Adv5-CMV-Cas9, (**D**) C57 Bl/6 murine strain injected with *Nf1/p53* AdV5-CMV-Cas9 (n = 19 biological replicates) and *Nf1/Cdkn2a* AdV5-CMV-Cas9 (n = 18 biological replicates), (**E**) Days to triple in tumor volume post tumor initiation across tumor genotype and mouse strains. (**F**) Comparison of tumor volume 10 days post tumor initiation across tumor genotype and mouse strains, each tumor normalized to its own tumor initiation volume (150-300 mm^3). Data presented as mean ± s.e.m. *P* values determined by Log-rank tests (in **A-D**), One-way Anova followed by Holm-Sidak correction (in **E** and **F**). *P* = 0.05

Fig. S2.

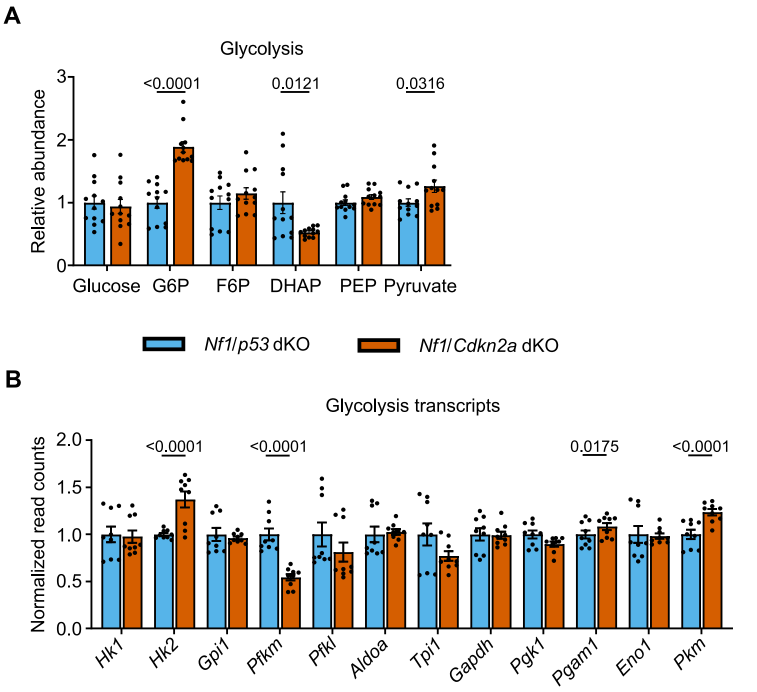

Differential tumor suppressor loss alters metabolite abundance and transcript levels in glycolysis. (**A-B**) Glycolysis pathway (**A**) relative metabolite abundances, normalized to *Nf1/p53* dKO average for each metabolite, G6P (glucose 6-phosphate), F6P (fructose 6-phosphate), DHAP (dihydroxyacetone phosphate), PEP (phosphoenolpyruvate). (**B**) RNAseq read count values normalized to *Nf1/p53* dKO average for each transcript. Data presented as mean ± s.e.m. *Nf1/p53* dKO (n=3) and *Nf1/Cdkn2a* dKO (n=3), each cell line run in quadruplicate (independent samples) (in **A**) and triplicate (independent samples) (in **B**). Unpaired t-tests (in **A**). Adjusted p-values determined by differential expression analysis followed by Benjamini and Hochberg's approach for FDR (in **B**). $P = 0.05$

Fig. S3.

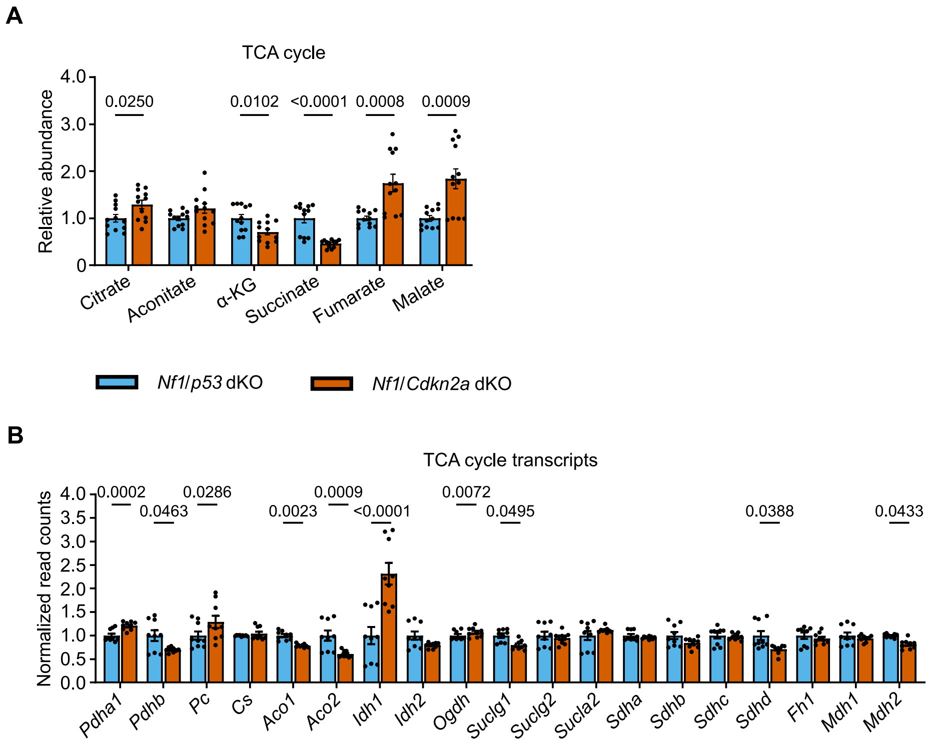

Differential tumor suppressor loss alters metabolite abundance and transcript levels in the TCA cycle pathway. (**A-B**) TCA cycle pathway (**A**) relative metabolite abundances, normalized to *Nf1/p53* dKO average for each metabolite. (**B**) RNAseq read count values normalized to *Nf1/p53* dKO average for each transcript. Data presented as mean ± s.e.m. *Nf1/p53* dKO (n=3) and *Nf1/Cdkn2a* dKO (n=3), each cell line run in quadruplicate (independent samples) (in **A**) and triplicate (independent samples) (in **B**). Unpaired t-tests (in **A**). Adjusted p-values determined by differential expression analysis followed by Benjamini and Hochberg's approach for FDR (in **B**). $P = 0.05$

Fig. S4.

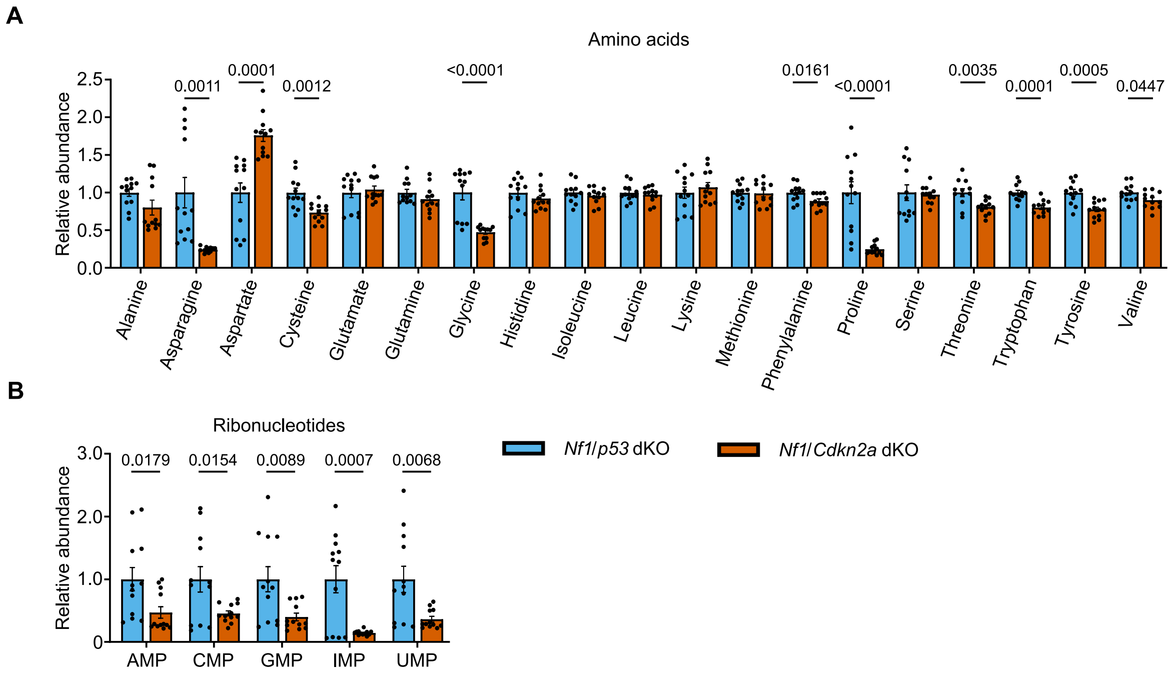

Differential tumor suppressor loss alters abundance of amino acids and ribonucleotides. (**A**-**B**) Relative metabolite abundances, normalized to *Nf1*/*p53* dKO average for each metabolite, (**A**) Amino acids and (**B**) Ribonucleotides, AMP (adenine monophosphate), CMP (cytidine monophosphate), GMP (guanosine monophosphate), IMP (inosine monophosphate), UMP (uridine monophosphate). Data presented as mean ± s.e.m. *Nf1*/*p53* dKO (n=3) and *Nf1*/*Cdkn2a* dKO (n=3), each cell line run in quadruplicate (independent samples) (in **A** and **B**). Unpaired t-tests (in **A** and **B**). *P* = 0.05

Fig. S5.

A

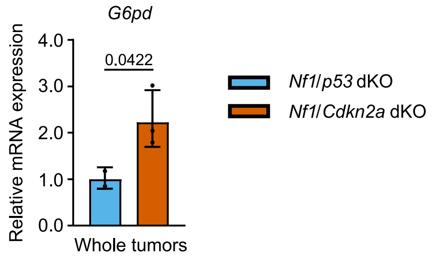

B

Genotype	Cell line	Inhibitor	IC50 (μM)	95% CI (μM)
Nf1/p53 dKO	*#1*	DHEA	220.6	204.2-240.2
	#2	DHEA	216.1	204.5-228.6
	#3	DHEA	260.1	231.3-316.2
Nf1/Cdkn2a dKO	*#1*	DHEA	98.5	95.4-101.7
	#2	DHEA	133.1	127.5-138.9
	#3	DHEA	91.5	80.7-103.1

C

Genotype	Cell line	Inhibitor	IC50 (nM)	95% CI (nM)
Nf1/p53 dKO	*#1*	Doxorubicin	1,201	1,063-1,459
	#2	Doxorubicin	1,037	827.0-1,431
Nf1/Cdkn2a dKO	*#1*	Doxorubicin	582.0	504.8-681.2
	#2	Doxorubicin	380.7	320.2-459.8

D

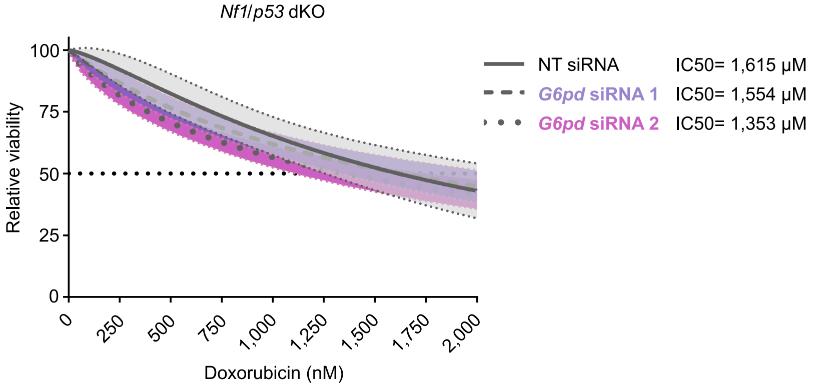

Response to DHEA and Doxorubicin are genotype dependent. (**A**) RT-qPCR verifying *G6pd* expression in whole tumor lysates (n=3). (**B**) Table showing IC50 results of individual *Nf1/p53* dKO and *Nf1/Cdkn2a* dKO cell lines treated with DHEA for 48 hours. (**C**) Table showing IC50 results of individual *Nf1/p53* dKO and *Nf1/Cdkn2a* dKO cell lines treated with doxorubicin for 48 hours. (**D**) Representative dose response curves of *Nf1/p53* dKO cells transfected with *G6pdx* targeted siRNAs and treated with doxorubicin 72 hours post transfection for 48 hours. Data presented as geometric mean and geometric standard deviation (in **A)**, two tailed unpaired t-tests (in **A**). IC50s and 95% confidence intervals determined using nonlinear regression, option "[inhibitor] vs normalized response-variable slope" in GraphPad Prism (in **B**, **C**, and **D**). Shaded areas represent 95% confidence intervals, line patterns represent specific siRNA treatments (in **D**).

Fig. S6.

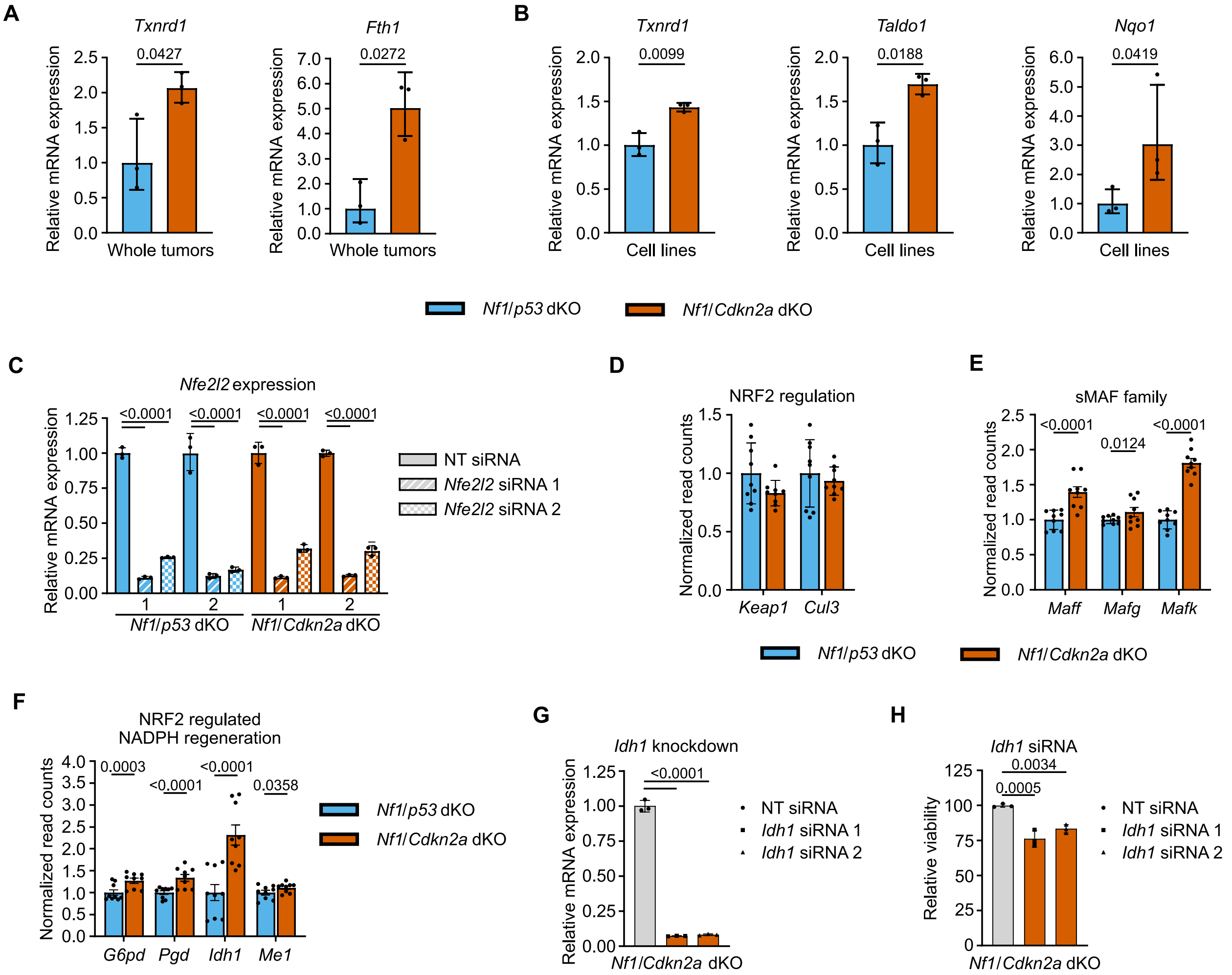

NRF2/PPP axis is the main NADPH producer, not Idh1. (**A**) RT-qPCR, verifying NRF2 target genes *Txnrd1* and *Fth1* in whole tumor lysates (n=3). (**B**) RT-qPCR, verifying expression levels NRF2 target genes *Txnrd1*, *Taldo1*, and *Nqo1* in primary tumor cell lines (n=3). (**C**) RT-qPCR verifying *Nfe2l2* (NRF2) knockdown 48 hours post transfection of *Nfe2l2* targeted siRNAs. (**D**-**F**) RNAseq, normalized read counts of (**D**) NRF2 transcription co-activators (**E**) NRF2 regulatory proteins and (**F**) NADPH producing genes regulated by NRF2. (**G**) RT-qPCR, verifying *Idh1* transcript knockdown 48 hours post transfection of *Idh1* targeted siRNAs (**H**) Relative viability of *Nf1*/*Cdkn2a* dKO cells 96 hours post transfection. Data presented as geometric mean and geometric standard deviation (in **A**, **B**, **C**, and **G**), two tailed unpaired t-tests (in **A** and **B**), One-way Anova followed by Holm-Sidak correction (in **C** and **G**). Data presented as mean ± SD, One-way Anova followed by Holm-Sidak correction (in **C** and **H**). Data presented as mean ± SD, *Nf1*/*p53* dKO (n=3) and *Nf1*/*Cdkn2a* dKO (n=3), each cell line run in triplicate (independent samples), Adjusted p-values determined by differential expression analysis followed by Benjamini and Hochberg's approach for FDR (in **D**, **E**, and **F**). $P = 0.05$

Fig. S7.

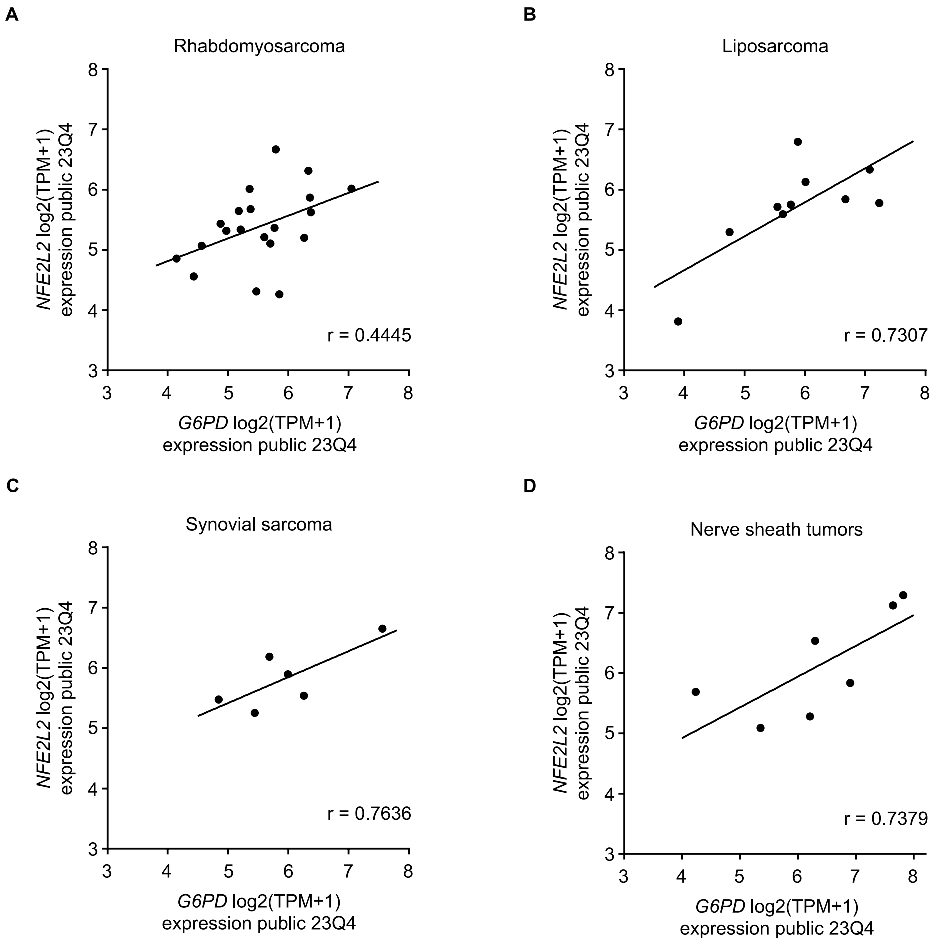

NRF2/G6PD axis extends to other sarcoma subtypes. (**A**-**D**) *NFE2L2* and *G6PD* expression in soft tissue sarcoma cell lines, (**A**) Rhabdomyosarcoma (n=20) (**B**) Liposarcoma (n=10) (**C**) Synovial sarcoma (n=6) (**D**) MPNST (n=7)**.** Analyzed using the Broad Cancer Dependency Map Project portal, each data point represents an individual cell line, Pearson correlation (r), (in **A**-**D**).

Table S1.

Primer/gRNA Name	Sequence (5' to 3')	Use
g*Nf1*	GGACATCTCCAAGGATGTGGTGG	gRNA targeting Nf1
g*Trp53*	GATGGTAAGGATAGGTCGGCGG	gRNA targeting Trp53
g*Ink4a/p16*	GGGCCGTGTGCATGACGTGC	gRNA targeting Ink4a/p16
g*Arf/p19*	ACTGTGAGGATTCAGCGCGC	gRNA targeting Arf/p19
Nf1 fw	TAGCAATTTTGGGGGAACGC	PCR for Indel Analysis
Nf1 rv	GTCAGAGCCTTTCAGTCATG	
Trp53 fw	TGCCGAACAGGTGGAATATC	PCR for Indel Analysis
Trp53 rv	CATCCTGACTGTGTGTAAACTAGGCCCC	
Ink4a/p16 fw	GTGTTTTCAGGGGTGTTCAATTCAT	PCR for Indel Analysis
Ink4a/p16 rv	CTTATTATGCACAGGCTCTGGAATG	
Arf/p19 fw	GGTGCCTCAACGCCGAA	PCR for Indel Analysis
Arf/p19 rv	CTGGTGGATGGGAGCGAAATAA	
B-actin fw	GGCTGTATTCCCCTCCATCG	RT-qPCR
B-actin rv	CCAGTTGGTAACAATGCCATGT	
B2M fw	GGTCTTTCTGGTGCTTGTCTC	RT-qPCR
B2M rv	GTTCAGTATGTTCGGCTTCCC	
G6pdx fw 1	CACCGTGGACGACATCCGAAA	RT-qPCR
G6pdx rv 1	GCAGGGCATTCATGTGGCT	
G6pdx fw 2	CTCCAATCAACTGTCGAACCA	RT-qPCR
G6pdx rv 2	TTGTCTCGATTCCAGATGGGG	
Nfe2l2 (NRF2) fw 1	CTTTAGTCAGCGACAGAAGGAC	RT-qPCR
Nfe2l2 (NRF2) rv 1	AGGCATCTTGTTTGGGAATGTG	
Nfe2l2 (NRF2) fw 2	TAGATGACCATGAGTCGCTTGC	RT-qPCR
Nfe2l2 (NRF2) rv 2	GCCAAACTTGCTCCATGTCC	
Idh1 fw 1	ATGCAAGGAGATGAAATGACACG	RT-qPCR
Idh1 rv 1	GCATCACGATTCTCTATGCCTAA	
Idh1 fw 2	CAGGCTCATAGATGACATGGTGG	RT-qPCR
Idh1 rv 2	CACTGGTCATCATGCCAAGGGA	
Glrx fw 1	CAACACCAGTGCGATTCAAGA	RT-qPCR
Glrx rv 1	GCAGAGCTCCAATCTGCTTCA	
Glrx fw 2	ATTAGATCACTGCATCCGCC	RT-qPCR
Glrx rv 2	ACAACACCAGTGCGATTCAA	
Txnrd1 fw	GGGTCCTATGACTTCGACCTG	RT-qPCR
Txnrd1 rv	AGTCGGTGTGACAAAATCCAAG	
Fth1 fw	GGCTGAATGCAATGGAGTGTG	RT-qPCR
Fth1 rv	GTGGTCACCCAGTTCTTTAATGG	
Taldo1 fw	GTAAAGCGCCAGAGGATGGAG	RT-qPCR
Taldo1 rv	CTCTTGGTAGGCAGGCATCT	
Nqo1 fw	GCCGAACACAAGAAGCTGGAAG	RT-qPCR
Nqo1 rv	GGCAAATCCTGCTACGAGCACT	

Guide RNA and RT-qPCR primer sequences utilized in this study. gRNA's are incorporated in the CRISPR/Cas9 adenovirus constructs to guide Cas9 to the desired gene targets.

Data S1.

GC & LC Mass spectrometry metabolomics data and statistical analysis. Data and statistics included in additional excel file (**Data_S1_metabolomics_data**). Data are normalized to *Nf1*/*p53* dKO average for each metabolite (row). Each column represents an individual sample ran (n = 12 /genotype). Means ± SEM are calculated in excel sheet for each metabolite. *P*-values are determined by unpaired T-Test in excel for each metabolite (row).
